# Let’s replay

**DOI:** 10.7554/eLife.43832

**Published:** 2018-12-18

**Authors:** Björn Rasch

**Affiliations:** Department of PsychologyUniversity of FribourgFribourgSwitzerland

**Keywords:** memory consolidation, olfactory targeted memory reactivation, slow-wave sleep, functional MRI, electroencephalography, ventromedial prefrontal cortex, Human

## Abstract

Reactivating brain activity patterns during sleep enhances memory performance the next day.

**Related research article** Shanahan LK, Gjorgieva E, Paller KA, Kahnt T, Gottfried JA. 2018. Odor-evoked category reactivation in human ventromedial prefrontal cortex during sleep promotes memory consolidation. *eLife* **7**:e39681. doi: 10.7554/eLife.39681

Sleep is crucial for learning and memory, but despite numerous studies, we still do not fully understand the underlying processes. Sleep protects our memories by keeping the outside world at bay, as we are processing less sensory information. Moreover, we have fewer internal conscious thoughts when we are asleep. But does sleep do more for our memories than simply protecting them from external and internal interference?

Current theories suggest that newly acquired memories are spontaneously reactivated or ‘replayed’ during deep or slow-wave sleep, which could act as additional relearning trials when the brain is ‘offline’ (Rasch and Born, 2013). Moreover, this could help integrate new memories into existing knowledge and so create long-term memories.

To study the spontaneous replay of memories during sleep researchers usually first measure the brain activity during learning and identify learning-related activity patterns. They then try to locate the same patterns during sleep. Several studies have successfully identified memory replay during sleep in rodents, birds and humans this way (Dave and Margoliash, 2000; Schönauer et al., 2017; Skaggs and McNaughton, 1996).

Another method is to teach participants new information paired with sensory cues, such as odors, sounds or words, and then attempt to reactivate a given memory by presenting the same cue when the participants are asleep. If the replay is relevant (that is, if replaying activity patterns during sleep is a function for memory formation), additional reactivation should enhance the memory performance. This actually works (Rasch et al., 2007; Rudoy et al., 2009; Schreiner and Rasch, 2015), and the technique is now known as targeted memory reactivation during sleep (Oudiette and Paller, 2013). However, so far it remained unclear whether re-exposure to cues during sleep indeed induces a replay of learning-associated brain activity patterns.

Now, in eLife, Jay Gottfried of Northwestern University and colleagues – including Laura Shanahan as first author – report evidence that certain activity patterns observed in the sleeping brain predict later memory performance (Shanahan et al., 2018). For their experiments, the researchers – who are based at the Northwestern and the University of Pennsylvania – combined brain scans and targeted memory reactivation. First, the volunteers had to memorize the location of various objects that belonged to different categories (animals, building, faces and tools). Then, fMRI scans were used to measure their brain activity and identify the brain patterns belonging to the four different categories (Figure 1). Afterwards, the participants learned to associate each category with a specific smell. The volunteers then spent the night inside an MRI scanner, during which they were exposed to two odors.

**Figure 1.**
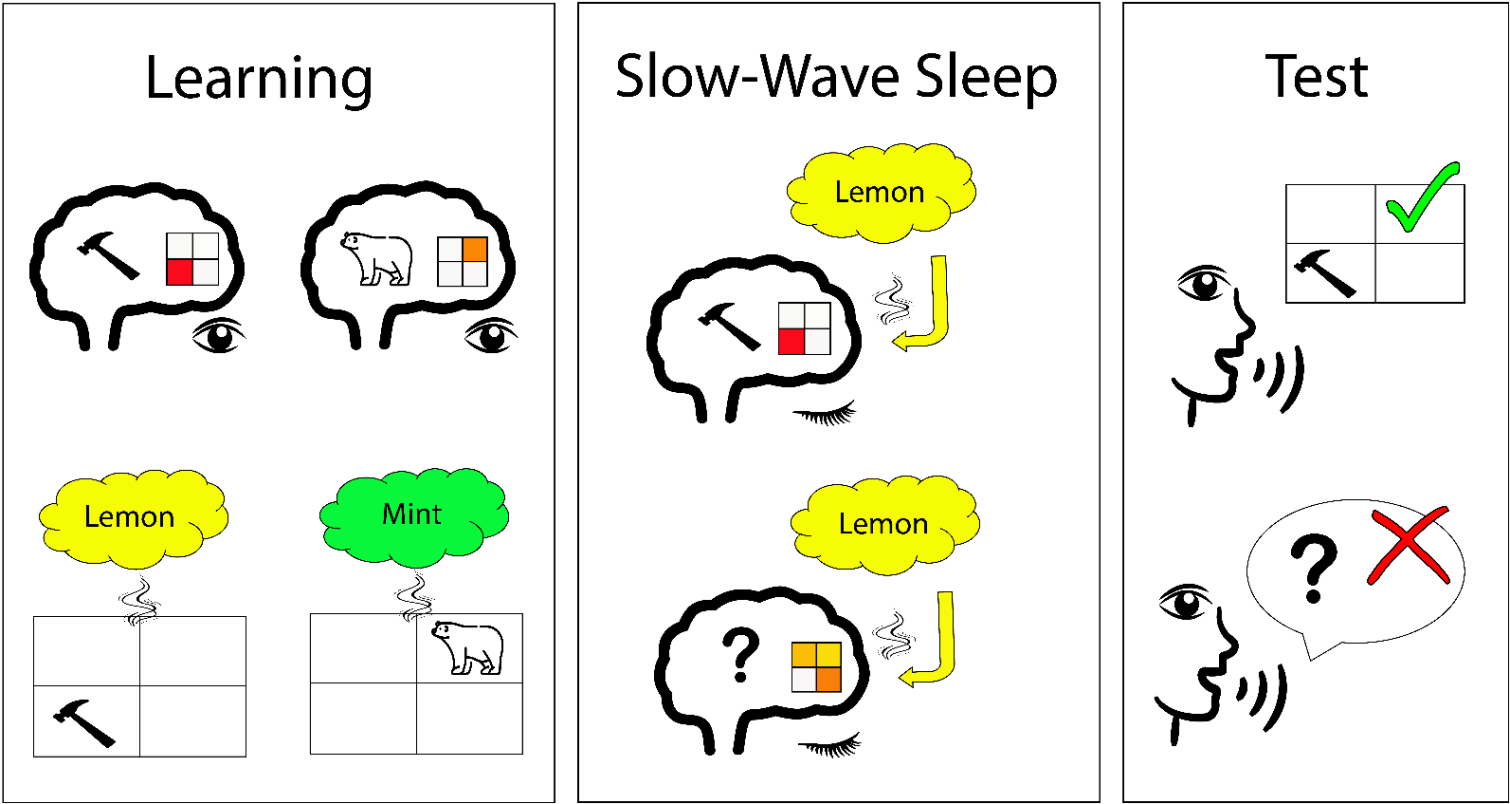
Odor-stimulated memory consolidation during sleep. Shanahan et al. first identified the brain activity patterns that are associated with different visual stimuli, including tools and animals (upper left); the 2x2 grid illustrates a certain pattern of brain activity. Afterwards (lower left), participants had to perform learning tasks while a specific smell (e.g., lemon) was presented for each visual category (e.g., tools). During slow-wave sleep, participants were exposed to the specific smell again. In a brain region called the ventromedial prefrontal cortex, the smell of lemon induced brain patterns similar to those observed for tools during the learning phase in some participants (upper middle). In other participants, the smell of lemon did not induce this pattern in this brain region (lower middle). The participants with a higher replay of brain patterns during sleep had a better memory performance after sleep (upper right) than those without (lower right).

Shanahan et al. were able to demonstrate that the cues presented again during sleep enhanced memory. Indeed, the participants were able to better recall the categories associated with the odor cues delivered during sleep. Moreover, during sleep, the scents induced a replay of the brain pattern associated with the specific stimulus category during prior learning.

To test whether this replay could predict memory performance, the researchers calculated the degree of overlap between the brain activity pattern observed during learning and after each odor presentation during sleep. The analyses revealed that the higher the overlap, the higher was the degree of memory replay induced by the odor. This applied in particular to a region in the brain important for retrieving old memories, called the ventromedial prefrontal cortex, where a higher replay index predicted a better memory performance. Furthermore, it started with the onset of the odor and persisted for several seconds, suggesting that the replay is tightly coupled to the odor presentation during sleep.

In sum, Shanahan et al. were able to show that memory replay induced by cues presented during sleep predicts the later memory performance in a specific brain area. This result is very important because it links for the first time the performance benefits of using cues to reactivate patterns during sleep and the detection of content-specific memory replay in the brain.

Some of the results that Shanahan et al. did not find are also important. For example, a brain area called the hippocampus did not show any predictive replay, contrary to studies in rodents, which have demonstrated such an activity in hippocampal cells (O'Neill et al., 2010; Ólafsdóttir et al., 2018). It remains to be seen whether this is due to methodological differences. Moreover, replay of activity patterns was generally observed only in visual areas and the left, frontal brain regions, which are also important for memory and other cognitive skills. However, neither of these regions was able to predict memory performance. Thus, our ability to predict performance by replay patterns appears to vary depending on the region of the brain.

In the future, studies using experimental manipulations of brain activity are needed to see if the replay of memory patterns during sleep is truly necessary for a better memory after sleep. And if yes, which brain areas are important? There is still a long way to go until we fully understand the mechanisms of memory consolidation during sleep.
